# Impact of Smoking Ban on Passive Smoke Exposure in Pregnant Non-Smokers in the Southeastern United States

**DOI:** 10.3390/ijerph15010083

**Published:** 2018-01-06

**Authors:** Julia C. Schechter, Bernard F. Fuemmeler, Cathrine Hoyo, Susan K. Murphy, Junfeng (Jim) Zhang, Scott H. Kollins

**Affiliations:** 1 Department of Psychiatry and Behavioral Sciences, Duke University Medical Center, 2608 Erwin Road, Durham, NC 27705, USA; scott.kollins@duke.edu; 2 Health Behavior and Policy, Virginia Commonwealth University, PO Box 980430, Richmond, VA 23298, USA; Bernard.Fuemmeler@vcuhealth.org; 3 Department of Biological Sciences, Center for Human Health and the Environment, North Carolina State University, Campus Box 7633, Raleigh, NC 27695, USA; choyo@ncsu.edu; 4 Department of Obstetrics and Gynecology, Duke University Medical Center, Box 91012, Durham, NC 27708, USA; susan.murphy@duke.edu; 5 Nicholas School of the Environment & Duke Global Health Institute, 308 Research Drive, Durham, NC 27701, USA; junfeng.zhang@duke.edu

**Keywords:** passive smoke exposure, environmental smoke exposure, secondhand smoke exposure, pregnancy, prenatal exposures, cotinine, biomarker, smoking, public policy, smoking ban

## Abstract

Prenatal passive smoke exposure raises risk for negative birth outcomes. Legislation regulating public smoking has been shown to impact exposure levels, though fewer studies involving pregnant women have been conducted within the U.S. where bans are inconsistent across regions. This study examined the effect of a ban enacted in the southeastern U.S. on pregnant women’s cotinine levels. Additional analyses compared self-reported exposure to cotinine and identified characteristics associated with passive exposure. Pregnant women (*N* = 851) were recruited prospectively between 2005 and 2011 in North Carolina. Sociodemographic and health data were collected via surveys; maternal blood samples were assayed for cotinine. Among non-active smokers who provided self-report data regarding passive exposure (*N* = 503), 20% were inconsistent with corresponding cotinine. Among all non-smokers (*N* = 668), being unmarried, African American, and less educated were each associated with greater passive exposure. Controlling for covariates, mean cotinine was higher prior to the ban compared to after, *F*(1, 640) *=* 24.65, *p* < 0.001. Results suggest that banning smoking in public spaces may reduce passive smoke exposure for non-smoking pregnant women. These data are some of the first to examine the impact of legislation on passive smoke exposure in pregnant women within the U.S. using a biomarker and can inform policy in regions lacking comprehensive smoke-free legislation.

## 1. Introduction

Comprehensive smoke-free legislation is inconsistent across the United States [1], and marked disparities remain regarding risk for passive smoke exposure [2]. Passive smoke exposure, which is considered exposure to secondhand smoke or environmental tobacco smoke, is especially concerning for pregnant women since it is linked to an array of negative perinatal outcomes, including preterm birth, low birth weight, restricted fetal growth, and Sudden Infant Death Syndrome [2,3,4,5,6]. Though bans have been shown to be effective in reducing passive smoke exposure in the general population [7,8,9] and pregnant women [10,11,12,13,14], the vast majority of these studies have been conducted outside of the U.S. [10,11,12,13,15,16]. This is particularly relevant in the southeastern U.S., a region lacking in comprehensive smoke-free legislation [1]. As the U.S. experienced a recent increase in rates of preterm birth, with the southeast having some of the highest rates in the country [17], identifying ways to protect pregnant women and their unborn children within the U.S. from the negative effects of passive smoke is of critical importance.

Notably, a potential barrier to measuring passive exposure during pregnancy is the reliance on self-report. Underreporting of smoking status (i.e., active smoker vs. non-smoker) has been observed among pregnant women [18,19], highlighting the importance of using additional corroborating measures such as biochemical validation. Though considered a valid measure of assessing passive exposure [20], relatively fewer studies have used such methods to measure self-reported passive exposure during pregnancy compared to self-report alone [6,21,22]. For example, in a recent meta-analysis including 24 studies examining the relationship between passive smoke exposure during pregnancy and preterm birth, only two studies included a biochemically-validated measure of passive exposure [2]. However, biochemical validation of self-reported exposure status may be especially critical when studying passive exposure during pregnancy, as many women may be unaware of their degree of exposure [23]. For instance, during 2007–2008, 40% of self-reported non-smokers in the U.S. had levels of serum cotinine, a metabolite of nicotine, indicating passive exposure [24], and serum cotinine levels suggestive of passive exposure have been found in 20–46% in representative samples of self-identified non-smoking pregnant women [21,22,25]. As no state in the southeastern region of the U.S. currently has a comprehensive smoke-free policy [1], biochemically validating self-reported passive exposure in pregnant women within this region may be particularly important for gaining a more accurate measurement of exposure during gestation. The current study examined cotinine levels from a sample of pregnant women recruited before and after the implementation of state legislation banning smoking in enclosed public areas including bars, restaurants, and hotels (North Carolina House Bill 2/S.L. 2009-27) to assess the impact of such policies on passive exposure during pregnancy.

In addition to policy-level factors, several individual-level characteristics such as fewer years of education [9,25,26], being unmarried [21], younger maternal age [26], and lower socioeconomic status [21,27] have been associated with greater passive exposure in both pregnant and non-pregnant populations. Further, disparities in passive smoke exposure based on culture, ethnicity, and race have been observed in the general population [9,28] and pregnant women [25,29,30]. For instance, Hawkins and colleagues [25] found that African-American pregnant women born within the U.S. were at higher risk for passive smoke compared to other ethnic minorities who emigrated to the U.S. Further, though less well examined, several studies suggest that mental health symptoms, such as self-reported depressive symptoms, increase risk for passive smoke exposure [31,32]. The current study sought to reexamine these previously identified individual-level characteristics in a diverse sample of women recruited prospectively during pregnancy.

The primary aim of the current study was to study the effect of a statewide smoking ban on prenatal passive smoke exposure, measured via plasma cotinine, in non-smoking pregnant women. The trajectory of cotinine levels in the weeks leading up to the ban and following implementation were also evaluated. In addition, we sought to examine the congruency between self-reported passive smoke exposure and cotinine levels and identify the sociodemographic and psychological factors associated with greater prenatal passive smoke exposure. Further, study participants were recruited from the southeastern U.S., an area lacking in comprehensive smoke-free policies and experiencing some of the highest rates of negative perinatal outcomes [17].

## 2. Materials and Methods

### 2.1. Participants

Participants were drawn from the Newborn Epigenetic STudy (NEST), a prospective study of pregnant women examining the effects of prenatal exposures on epigenetic profiles and child development. Enrollment procedures are similar to those described previously [33,34]. Briefly, between 2005 and 2011, pregnant women were recruited through prenatal clinics serving Duke University Hospital and Durham Regional Hospital Obstetric facilities in Durham, North Carolina. Inclusion criteria included women ≥18 years of age that spoke English and/or Spanish. All participants provided written informed consent. The study was approved by the Duke University Institutional Review Board (Pro00014548) and conducted in accordance with the Declaration of Helsinki and guidelines established by the Federal Government for the protection of human subjects.

At the time of enrollment during their prenatal visit, women completed self-report or interview-administered questionnaires, which included questions regarding sociodemographic characteristics (e.g., age, race, level of education, marital status) and maternal health and lifestyle factors (e.g., cigarette smoking status). Questions from the Perceived Stress Scale [35] and Center for Epidemiological Studies Depression Scale [36] were included at certain waves of recruitment. Questionnaires were offered in both English and Spanish. Trained personnel abstracted parturition data from medical records after delivery, including pregnancy hypertension, preeclampsia, gestational diabetes, and parity.

Recruitment for the NEST study occurred in two waves. During wave 1 (2005–2008), 1101 women were approached to participate in the NEST study, and 940 (85%) were enrolled [37]. Based on the study aims at the time, ~200 of the women initially enrolled during wave 1 reported smoking during pregnancy. Women in wave 2 were recruited from 2009–2011, and there was no enrollment criterion regarding smoking status. In addition, the wave 2 survey was expanded to include a wider range of self-reported smoking behaviors, including questions about passive smoke exposure, and measures assessing depression and anxiety. During this wave, 2548 women were approached to participate, and 1700 (66.6%) were enrolled [38].

The current analyses were comprised of 851 women (mean age = 28.01 years, *SD* = 5.71 years) from the larger NEST study for whom plasma blood samples were available (see below) and had been assayed for cotinine. Of these women, 184 from wave 1 and 12.6% (*N* = 75) of the participants in wave 2 were recruited prior to the 2010 ban; the remaining participants from wave 2 (*N =* 593) were recruited after the ban. The subset of plasma samples selected for assay was based on whether women had participated in later follow-up studies examining the long-term impact of prenatal smoke exposure. These women were more likely to be African American (53% in the current sample vs. 39% in the full NEST sample), less likely to identify as Hispanic (0.04% in the current sample vs. 24% in the full NEST sample), and slightly more educated (82% attended at least some college in the current sample vs. 67% in the full NEST sample); thus, the current sample was not representative of the full NEST sample. Notably, the current sample did not differ significantly from the full NEST sample with regard to rates of self-reported smoking during pregnancy. The current sample also did not differ across recruitment waves except with regard to cotinine levels, with women recruited during wave 1 having higher cotinine levels (wave 1: mean = 22.68, *SD* = 58.26; wave 2: mean = 14.24, *SD* = 43.45).

### 2.2. Smoking Ban

The North Carolina General Assembly passed legislation (North Carolina House Bill 2/S.L. 2009-27) banning smoking in enclosed public areas including bars, restaurants, and hotels in May 2009, and the law went into effect on 2 January 2010. The implementation process included the launching of a website that provided education about the law; holding information sessions and distributing packets and resources to affected businesses; and a phone and online system for reporting violations [39]. The ban was enforced by the Tobacco Prevention and Control Branch of the North Carolina Department of Health and Human Services, and weekly compliance and violation reports were posted online; however, the number of reported complaints declined precipitously within two months following implementation of the ban and has remained low (i.e., approximately 10 complaints per month as of November 2013), suggesting a high level of compliance with the ban [39].

### 2.3. Cotinine Collection and Assay Procedures

Plasma blood samples were collected from women during pregnancy (mean gestational age = 17.30 weeks, *SD* = 11.28 weeks). Assays were completed at the Exposure Biology and Chemistry Lab at Duke University. Cotinine was measured using a high-performance liquid chromatography with tandem mass spectrometric detection (HPLC-MS-MS) method, a highly sensitive assay designed to measure levels of environmental smoke exposure with a limit of detection of 0.05 ng/mL [40,41,42] and a reproducibility >94% [40,41,42]. The HPLC-MS-MS system consisted of a TSQ Quantum Access Max™ triple quadrupole mass spectrometer (Thermo Fisher Scientific, San Jose, CA, USA) coupled to a Thermo Fisher Accela 1250 liquid chromatograph (Thermo Fisher Scientific, San Jose, CA, USA). To conduct the analyses, a 0.2-mL plasma sample was placed in a 1.5-mL plastic micro tube, mixed with 20 µL of 100 ng/µL of cotinine-D3 (internal standard) and 1 mL of methanol, and vortexed to mix thoroughly. After centrifugation at 16,000× *g* for 10 min, the supernatant was transferred to a conical-bottom glass centrifuge tube. After the solvent evaporated, the residue was reconstituted in 200 µL of acetonitrile/water (15%:85%), vortexed, and then centrifuged at 16,000× *g* for 10 min. A 20-µL aliquot of the supernatant was injected into the HPLC-MS-MS system. Chromatographic separation was achieved on a Phenomenex Luna (Torrance, CA, USA) 3 µ C18 (50 × 2 mm) column using an isocratic mobile phase elution (water/acetonitrile containing 0.1% formic acid: 85/15) at a flow rate of 200 µL/min. The mass spectrometer was operated in a positive electrospray ionization (ESI) mode. The capillary temperature and vaporizer temperature were at 350 °C and 300 °C, respectively. The ion spray voltage was set to 3000. Nitrogen sheath and auxiliary gases were set to 40 and 15 arbitrary units, respectively. The ion pairs of m/z 177/98, 180/80 were used to monitor cotinine and cotinine-d3.

### 2.4. Assessment of Smoking Status Based on Cotinine

Cotinine cut-points were based upon those recommended by Benowitz and colleagues [43] and used previously in studies of passive smoke exposure during pregnancy [25,44]. Specifically, unexposed non-smokers were defined as women with cotinine levels below 1 ng/mL, passive exposure was defined as cotinine levels ranging from 1–3 ng/mL, and active smoking was defined as cotinine levels greater than 3 ng/mL. Due to non-normality, cotinine values were natural log-transformed for analyses that assume normally distributed data. As log transformations for values of zero are undefined, a constant was added to all cotinine values prior to the log transformation.

### 2.5. Assessment of Smoking Status Based on Self-Report

Women indicated whether they had smoked at all during pregnancy at the time the plasma sample was collected on their questionnaire. Women were classified as self-reported “non-smokers” if they indicated that they had not smoked at any point during pregnancy, and were classified as self-reported “smokers” if they reported smoking at any time during pregnancy. During wave 2, women were also asked to report on their current passive smoke exposure at work and within their home. Specifically, women were asked to report the current smoking habits of all individuals inside of their home and indicate whether, “No one smokes anywhere inside the house,” “Smoking is allowed in some rooms in the house,” or “Smoking is permitted anywhere inside the house.” Similarly, women were asked to report on current smoking habits at their place of work if they worked outside of the home. Response options included, “No one smokes anywhere inside my place of work,” “Smoking is allowed in designated areas inside,” or “Smoking is freely permitted inside the workplace.” Responses were coded as “exposed” (i.e., smoking is allowed in part or all of my home/work) or “not exposed” (i.e., no one smokes anywhere in my home/work) to passive smoke.

### 2.6. Assessment of Maternal Prenatal Stress and Depression

Maternal prenatal stress was measured using the Perceived Stress Scale (PSS) [35]. The PSS is a 10-item self-report questionnaire that measures an individual’s evaluation of the stressfulness of situations occurring within the past month. For each question, scores range from 0 (never) to 4 (very often). Average scores for women on this measure are 13.7 (*SD* = 6.6), with scores of 20 or higher indicating high stress [45]. The PSS has been shown to have good reliability and validity across samples [35].

Maternal prenatal depression was measured using the Center for Epidemiologic Studies Depression Scale (CES-D), a widely used assessment of depressive symptoms frequently utilized in studies with pregnant women [46,47]. Respondents are asked to indicate how often they have experienced each symptom with response options ranging from 0 (rarely/none of the time) to 3 (most/all of the time). Scores range from 0–60, and a score of 16 is often used as the cut-off score for depression. The CES-D has been found to have good sensitivity and high internal consistency [36,48].

### 2.7. Data Analysis

The primary objectives of the study were addressed as follows. First, women’s self-reported passive smoke exposure status and their plasma cotinine levels were compared to assess the congruency between women’s self-report and cotinine concentrations. Second, to determine sociodemographic and psychological characteristics associated with passive exposure during pregnancy, a series of separate Analysis of Variance (ANOVA) and multiple linear regression analyses were run using the sample of women identified as non-smokers based on cotinine values (i.e., ≤3 ng/mL). Analysis of Covariance (ANCOVA) procedures were conducted to assess the effect of a 2010 smoking ban on passive exposure during pregnancy (enacted on 2 January 2010) for women identified as non-smokers based upon cotinine levels. In addition, a regression discontinuity design was used to compare the pattern of cotinine levels in non-smoking women in the days leading up to and following the implementation of the ban. Demographic variables were explored as potential covariates, and variables significantly associated with the dependent variable were included in relevant models. All analyses were conducted using SPSS version 24 (SPSS Inc., Armonk, NY, USA).

## 3. Results

### 3.1. Sample Characteristics

Demographic characteristics are presented in Table 1. Given the small samples of women in the Hispanic and Other categories (3.6% and 4.3%, respectively) for race/ethnicity, these groups were combined. Within the current sample, 6.2% had hypertension during pregnancy, 4.8% had preeclampsia, and 5.8% had gestational diabetes. Parity for the sample ranged from zero (i.e., currently pregnant with first child at time of enrollment) to the ninth pregnancy (mean = 1.11). PSS scores (*M* = 14.03, *SD* = 6.67) fell slightly above the average scores on this measure, though they were still within one standard of deviation for women [45]. CES-D scores (*M* = 12.06, *SD* = 8.51) fell below the depression cut-off score.

As can be seen in Table 1 and Table 2, over half of the women identified as Black/African American, and this group also had the highest cotinine levels compared to Caucasian and Hispanic/Other groups. Most of the women were married at the time of their study enrollment, and these women had the lowest levels of cotinine in the current sample and among non-smokers (identified by cotinine levels). Though the sample were largely college-educated, over a third had not attended or graduated from college, and these women had higher cotinine levels compared to those who had attended at least some college. As illustrated in Table 2, across all demographic variables with sufficient sample sizes (i.e., *N* > 1), mean cotinine levels declined after the ban. Analyses of cotinine levels before and after the ban based upon sociodemographic characteristics are presented below.

### 3.2 Comparison of Self-Reported Passive Smoking Status and Cotinine Levels

Mean plasma cotinine levels were 16.07 (*SD* = 47.15) for the full sample (*N* = 851), 62.05 (*SD* = 81.66) for self-reported smokers (*N* = 189), and 1.99 (*SD* = 9.26) for self-reported non-smokers (*N* = 631). Of the 851 women, 62.6% (*N* = 533) had cotinine values below 1 ng/mL, indicating minimal to no smoke exposure; 15.8% (*N* = 135) had cotinine values between 1 and 3 ng/mL, indicating passive smoke exposure; and 21.5% (*N* = 183) had cotinine values of 3 ng/mL or greater, indicative of active smoking. Both cotinine and self-report information regarding passive smoke during pregnancy were available for 503 non-smoking (i.e., cotinine ≤3 ng/mL) women. Among the 433 women who reported that they had not been exposed to passive smoke, 51 (11.8%) were considered “under-reporters” and had plasma cotinine levels indicative of passive smoke exposure. Among the 70 women who reported that they had been exposed to passive smoke exposure, 50 (71.4%) were considered “over-reporters” and had plasma cotinine levels that did not indicate passive smoke exposure (Table 3). Overall, 79.9% (i.e., 402/503) of the self-reported responses regarding passive smoke exposure were congruent with cotinine levels (i.e., women who reported “no exposure” had cotinine levels indicative of no exposure; women who endorsed passive exposure had cotinine levels consistent with passive exposure), while 20% (101/503) of responses were incongruent with cotinine values (i.e., women were either over- or under-reporters of their exposure). Cohen’s Kappa was used to assess the agreement between self-reported passive exposure and cotinine categories. Results suggested poor agreement (*K* = 0.17, *p* < 0.001) between the self-report and cotinine categories. Additional separate sensitivity analyses excluding women who reported quitting during pregnancy and lowering the threshold to indicate non-smoking (i.e., cotinine levels <1 ng/mL) did not affect the results (data not presented).

### 3.3. Cotinine Levels and Characteristics of Non-Smoking Pregnant Women

A series of ANOVAs were run to determine the sociodemographic (Table 2) and psychological factors associated with greater passive smoke exposure among non-smoking pregnant women (i.e., cotinine ≤3 ng/mL; *N* = 668). Cotinine values differed significantly by race, *F*(2, 665) = 3.37, *p* = 0.04. African Americans had the highest levels of cotinine followed by Caucasians, and Hispanic/Other women had the lowest levels. Post-hoc comparisons using the Tukey Honest Significant Difference (HSD) test indicated a significant difference between African Americans and Hispanic/Other women. Marital status was also significantly associated with passive exposure, *F*(4, 644) = 3.54, *p* = 0.007, with married women having the lowest exposure and those divorced/separated or living with a partner having the highest levels. Post-hoc comparisons revealed a statistically significant difference between the married and never married groups. Lower educational attainment was significantly associated with higher levels of passive smoke exposure, *F*(3, 646) = 2.92, *p* = 0.03. Post-hoc comparisons revealed a statistically significant difference between the women who had graduated college and those who had a high school diploma/GED (General Equivalency Degree). Notably, women who had not attended college had significantly higher cotinine levels compared to those who had attended some college or were college graduates, *t* = (648) 2.38, *p* = 0.017. Age at the time of pregnancy was not associated with passive smoke exposure, *F*(2, 659) = 2.23, *p* = 0.11, though younger women had higher mean levels of cotinine compared to older women. Women with a greater number of previous pregnancies also had higher levels of cotinine; however, parity was not significantly associated with cotinine levels, *F*(4, 652) = 1.68, *p* = 0.15.

Multiple linear regression analyses were run to assess whether stress or depressive symptoms were associated with passive smoke exposure. Control variables (i.e., race, educational attainment, wave of recruitment, and marital status) were entered into block 1, and the predictor variable was entered into block 2. In separate analyses, neither PSS scores, *β* = −0.10, *t*(224) = −1.40, *p* = 0.16, nor CES-D scores, *β* = 0.01, *t*(467) = 0.18, *p* = 0.86, were significantly associated with passive smoke exposure in non-smokers.

### 3.4. Effect of the Smoking Ban in Public Spaces in Non-Smoking Pregnant Women

**Figure 1:**
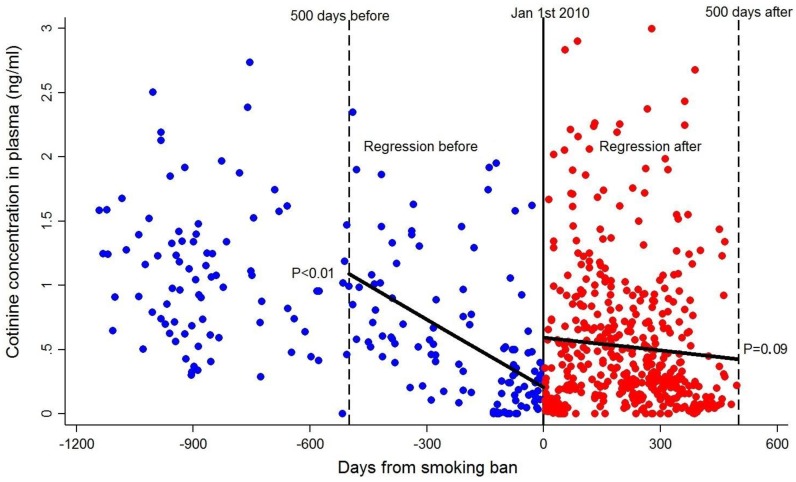
Levels of cotinine among non-smoking (i.e., cotinine levels ≤3 ng/mL) pregnant women before (*N* = 200, mean = 0.78, *SD* = 0.60) and after (*N* = 468, mean = 0.52, *SD* = 0.55) implementation of the 2010 smoking ban. Data are plotted from the exact day (2 January 2010) that the ban was implemented. Results from the regression discontinuity analyses are also displayed. Restricting the data to 500 days before the ban (*N* = 114) and 500 days after the ban (*N* = 464), results indicate a significant decline in cotinine values in the days prior to implementation of the smoking ban and a non-significant decline following the ban.

For pregnant women identified as non-current smokers (i.e., ≤3 ng/mL, *N* = 668), cotinine levels were analyzed before (*N* = 200) and after (*N* = 468) the 2010 ban (Figure 1) using ANCOVA. Race, educational attainment, wave of recruitment, and marital status were included as covariates. Results indicated that cotinine was significantly higher prior to the ban (mean = 0.78, *SD* = 0.60) compared to after (mean = 0.52, *SD* = 0.55), *F*(1640) = 24.65, *p* < 0.001, Cohen’s *d* = 0.45. Sensitivity analyses restricted to 500 days before and after the ban did not alter these findings. In addition, using a regression discontinuity design and controlling for aforementioned covariates, the results also showed a significant negative decline in smoke exposure 500 days ahead of the ban, *β* = −0.47, *t*(113) = −2.67, *p* = 0.009, and lower but steady decline in exposure 500 days after the ban at a trend level, *β* = −0.08, *t*(443) = −1.70, *p* = 0.09.

## 4. Discussion

Among non-smoking pregnant women in the southeastern U.S., results indicated a significant decrease in plasma cotinine levels leading up to and following a statewide ban restricting smoking in public spaces. In addition, several individual-level characteristics were associated with increased risk for passive smoke exposure, including being African American, unmarried, and lower educational attainment. Lastly, approximately 20% of women’s self-reported passive exposure was incongruent with plasma cotinine levels.

These results have important implications as rates of smoking during pregnancy differ between the U.S. and other countries [49], with a greater number of studies examining passive smoke exposure in pregnant women conducted outside of the U.S. [10,11,12,13,15,16]. For example, both Puig and colleagues [11] and Franchini et al. [12] found a significant decrease in cotinine levels collected from cord blood in non-active smokers following the implementation of smoke-free policies in Spain and Italy, respectively. Within the U.S., Markowitz et al. [50] used data from the Pregnancy Risk Assessment Monitoring System (PRAMS) collected from 29 states and New York City to examine how several different types of tobacco control policies, including comprehensive smoking bans, impacted birth outcomes. Results indicated that banning smoking in restaurants was associated with improved birth outcomes for older, better-educated, higher socioeconomic status (SES) women, but reduced effectiveness for teenage mothers. Notably, the PRAMS data includes limited information regarding secondhand smoke exposure and the authors were unable to assess the effect of these policies on passive smoke exposure in pregnancy [50]. Page and colleagues [14] examined rates of self-reported smoking, preterm births, and babies born with low birth weight before and after a citywide smoking ban in Colorado, and compared these rates to a comparable city without smoke-free legislation. Results indicated a significant reduction in self-reported smoking and preterm births, and a reduced odds of having a low birth weight baby, though results were not statistically significant. Notably, this study did not differentiate between active smokers and women exposed to passive smoke exposure in pregnancy. In a another prospective study with self-reported non-smoking pregnant women in New York City, Hawkins and colleagues [25] collected blood samples from African American, Caribbean American, and Black Hispanic pregnant women. Results indicated that nearly half of the sample had detectable cotinine levels and 46.8% of the sample had levels consistent with passive smoke exposure, even after implementation of a comprehensive tobacco control program.

The current study builds upon these findings by examining cotinine levels indicative of passive smoke exposure in a sample of pregnant women within the southeastern U.S. To our knowledge, this is the first study examining the association between the implementation of a smoking ban on passive smoke exposure during pregnancy within this region, and is one of only a few to examine the impact of smoking bans on passive exposure during pregnancy within the U.S. Results appear to support a link between a policy aimed at regulating tobacco and a biomarker of passive smoke exposure, and also suggest that passive smoke exposure decreased in the days leading up to the implementation of the ban for pregnant women who were non-active smokers. Notably, despite the apparent effectiveness of the ban, results suggest that even after the implementation of smoke-free legislation, over a third of the sample had been exposed to smoke—either actively or passively—during pregnancy using a biochemical marker. Though this represents an improvement compared to the approximately 48% (i.e., 124/259) exposed to smoke prior to the ban, these findings underscore the relevance of gestational smoke exposure as a public health concern. Further, these results are important given the multitude of negative sequelae associated with perinatal smoke exposure [2,3,4,5] as well as the long-term positive child health outcomes associated with smoking bans [51]. In particular, in the context of a rising preterm birth rate, the current study provides policy-makers with needed data to bolster support for comprehensive smoke-free policies within the southeast and other regions in the U.S. with similarly lacking legislation.

The current results replicate previous findings using biochemical validation of smoking status rather than self-report alone [10,11,12]. These findings are important given the previously discussed discrepancy and limitations of assessing exposure based solely on self-report. Specifically, the current study corroborates previous findings that as many as 20% of non-smoking women may be inaccurate in their assessment of their level of passive exposure during pregnancy [21,22]. Notably, the majority of women identified as having been passively exposed in the current study did not endorse such exposure. Thus, these results reemphasize that many women may be unaware of or misreport their level of passive exposure during pregnancy [23,25].

The current study also replicates previous results that certain demographic characteristics—including race, marital status, and education level—are associated with higher levels of passive smoke exposure during pregnancy. In particular, consistent with previous literature [25,28,29], African American women had the highest level of passive smoke exposure compared to other racial groups, and these results remained significant when controlling for SES-related demographic factors (i.e., educational attainment, marital status). Higher levels of cotinine in non-pregnant African American samples have been attributed to several factors, including a greater density of tobacco retail outlets located in areas with racial/ethnic minority populations [52], a history of targeted marketing and promotion of tobacco products in African American publications [53], differences in nicotine metabolism [54], and relative preferences for mentholated cigarettes [55], which some studies have associated with higher nicotine levels [56]. Replicating these findings in a sample of pregnant women is especially important as African Americans experience a disproportionate rate of disease burden compared to Caucasians, and the current results indicate that disparities in the effects of smoke exposure begin in utero even among non-active smokers.

The current results should be interpreted in the context of some limitations. Data were collected over two waves of a longitudinal study that evolved over time; thus, certain measures were not collected on all women, including questions regarding depression and stress, or in the same level of detail, including questions regarding timing of pre-pregnancy and prenatal smoking. For example, only women in wave 2 were asked to provide self-report data about their level of passive exposure at home and work. Thus, we were only able to compare cotinine values to self-reported passive exposure on a subsample of women, and the majority of these women were enrolled after the implementation of the smoking ban. In addition, though questions asked about different levels of passive smoke exposure at home and at work, small sample sizes limited our ability to compare cotinine levels across these different groupings and were thus examined as a dichotomous variable (any passive smoke exposure vs. no passive smoke exposure). Due to funding limitations, only plasma samples from women who participated in later follow-up studies were analyzed for cotinine. As noted above, the subsample included in the current analyses differed from the larger NEST study sample in several ways. Relatedly, though the sample was composed of a majority of African American women and was diverse with regard to SES-related factors (e.g., education), relatively few women identified as Hispanic/Latino or other races/ethnicities, limiting our ability to clarify whether other subgroups may be at high risk for passive smoke exposure during gestation. Future studies will also want to examine the role of dwelling type (e.g., apartment vs. home, rental unit vs. owned home); this level of analysis was not possible with our data. Overall, these findings may not be as generalizable to ethnically diverse, densely urban areas.

A notable caveat to this study is that we examined self-report in relation to cotinine levels as measured in plasma. The half-life of cotinine in plasma is between 48 and 72 h. Some have proposed the use of hair to measure cotinine, which may reflect exposure over longer periods of time (months) [57]. Therefore, comparing self-report to cotinine assayed from various biological matrices is ripe for future investigation. Another consideration in the current study are the number of women who were identified as “over-reporters” (i.e., reported being exposed to passive smoke but had cotinine levels indicative of no smoke exposure). The rate of “over-reporters” is higher than other studies that have compared self-reported smoking status during pregnancy to a biomarker [22,58,59]. This may be reflective of women who are vigilant to the potential harms in their environment, especially while pregnant, as well as to the wording of the questions regarding passive smoke exposure (e.g., not asked to specify how recently others had smoked around them). Notably, while other studies have examined the concordance of self-report of smoking in relation to cotinine levels among self-reported non-smokers [22,58,59], this study is unique in that we examined self-report of passive smoke exposure among non-smokers categorized based on their cotinine levels (i.e., ≤3 ng/mL). Given the unique nature of this study, it is difficult to compare our findings to those of others; however, the present results reemphasize that the misclassification of passive smoke exposure among non-smokers (both over- and under-reporting) is a significant issue when using self-report.

The study is also notable for several strengths. In particular, using a biochemically validated measure of nicotine exposure allowed for the examination of the impact of the smoking ban and the corroboration of self-report measures in a sample of pregnant women using an objective outcome measure. Notably, one factor influencing the interpretation of the present results are the cut-points used for distinguishing among smoking categories. Several different cut-points have been proposed to indicate active and passive smoke exposure in pregnancy [57]. For example, Florescu and colleagues provided the reference value of 10 ng/mL to distinguish between active and passive smoking; however, additional analyses (not presented) using this proposed cut point did not change the impact of the ban in the current sample. The cut-points chosen for the current analyses were based upon those recommended by Benowitz and colleagues [43], as well as those used in a similar study examining passive smoke exposure during pregnancy [25].

Other strengths of the current study include prospective data collection, thus avoiding difficulties related to retrospective reporting. In addition, the sample was larger than similar studies that have examined passive smoke exposure during pregnancy using a biological marker of exposure [4,25,60]. The current study adds to the growing literature studying the impact of smoking bans within the U.S. and provides novel data regarding the impact of a smoking ban in an area of the U.S. with limited smoke-free legislation. Taken together, the findings from this regional cohort are in line with a growing set of studies suggesting that smoke-free laws may have “spillover” effects and reach beyond reducing adult smoking. Such policies may also help protect those vulnerable to secondhand smoke exposure, including infants and children [51].

In addition to the policy implications, these findings are informative for medical and mental health providers working with pregnant women. Specifically, providers will likely want to be aware of current smoking legislation within the region that they practice and attend to several individual characteristics of their patients including race/ethnicity, marital status, and educational attainment when considering who may be at the greatest risk for passive tobacco smoke exposure. In addition, results indicate that simply asking women about their level of exposure may not be sufficient for ascertaining accurate information. Thus, providers might want to take additional time to discuss possible prevention efforts (e.g., encouraging women to speak to their employers about reducing smoke exposure in the workplace, limiting smoking by partners within the home) with women at higher risk for exposure.

## 5. Conclusions

In summary, the present results indicate that banning smoking in public spaces may reduce passive smoke exposure in non-smoking pregnant women. The current study also replicates previous findings regarding individual level characteristics associated with gestational passive smoke exposure and underscores that many women may be unaware of their level of exposure. Replicating these findings within a U.S. sample in the southeast is especially critical for policy-makers and may be helpful for increasing wider-spread smoking policies within this and other regions lacking comprehensive smoke-free legislation.

## Figures and Tables

**Table 1:** Sociodemographic characteristics for full sample, non-smokers, and non-smokers before and after the 2010 smoking ban.

	Full Sample	All Non-Smokers	Non-Smokers Pre-Ban	Non-Smokers Post-Ban
	*N* (%)	*N* (%)	*N* (%)	*N* (%)
Race				
African American	452 (53.1)	336 (50.3)	89 (44.5)	247 (52.8)
Caucasian	332 (39.0)	274 (41.0)	99 (49.5)	175 (37.4)
Hispanic/Other	68 (8.0)	58 (8.7)	12 (6.0)	46 (9.8)
Marital Status				
Never married	263 (30.9)	184 (27.5)	48 (24.0)	136 (29.1)
Married	380 (44.6)	346 (51.8)	111 (55.5)	235 (50.2)
Living with partner	138 (16.2)	91 (13.6)	28 (14.0)	63 (13.5)
Divorced/separated	28 (3.3)	19 (2.8)	10 (5.0)	9 (1.9)
Other	16 (1.9)	9 (1.3)	1 (.5)	8 (1.7)
Education				
<High school	120 (14.1)	61 (9.1)	10 (5.0)	51 (10.9)
High school/GED	177 (20.8)	125 (18.7)	45 (22.5)	80 (17.1)
Some college	198 (23.2)	145 (21.7)	50 (25.0)	95 (20.3)
College graduate	332 (39.0)	319 (47.8)	93 (46.5)	226 (48.3)
No college	297 (34.9)	186 (27.8)	55 (27.5)	131 (28.0)
Any college	5030 (62.2)	464 (69.5)	143 (71.5)	321 (68.6)
Parity				
0	320 (37.6)	270 (40.4)	58 (29.0)	212 (45.3)
1	288 (33.8)	222 (33.2)	71 (35.5)	151 (32.3)
2	140 (16.4)	104 (15.6)	44 (22.0)	60 (12.8)
3	48 (5.6)	34 (5.1)	11 (5.5)	23 (4.9)
≥4	45 (5.3)	27 (4.0)	12 (6.0)	15 (3.2)

Note. Non-smokers identified by cotinine values ≤3 ng/mL. Non-transformed cotinine values are presented. GED = General Equivalency Degree.

**Table 2:** Cotinine values for full sample, non-smokers, and non-smokers before and after the 2010 smoking ban.

	Full Sample	All Non-Smokers	Non-Smokers Pre-Ban	Non-Smokers Post-Ban
	Cotinine (Mean, *SD*)	Cotinine (Mean, *SD*)	Cotinine (Mean, *SD*)	Cotinine (Mean, *SD*)
Race				
African American	19.21 (53.25)	0.68 (0.65)	0.92 (0.65)	0.59 (0.62)
Caucasian	13.65 (39.57)	0.52 (0.48)	0.63 (0.51)	0.46 (0.45)
Hispanic/Other	6.93 (34.87)	0.50 (0.58)	0.91 (0.73)	0.39 (0.48)
Marital Status				
Never married	21.82 (55.86)	0.67 (0.65)	0.85 (0.65)	0.61 (0.64)
Married	6.61 (30.11)	0.52 (0.50)	0.68 (0.54)	0.44 (0.46)
Living with partner	19.70 (42.81)	0.72(0.69)	1.03 (0.77)	0.58 (0.61)
Divorced/separated	29.54 (59.33)	0.77 (0.45)	0.81 (0.34)	0.74 (0.56)
Other	57.15 (91.21)	0.61 (0.67)	0.01 (--)	0.68 (0.68)
Education				
<High school	42.53 (76.19)	0.73 (0.75)	0.75 (0.50)	0.73 (0.79)
High school/GED	24.41 (60.37)	0.72 (0.64)	0.99 (0.67)	0.57 (0.57)
Some college	16.79 (41.31)	0.62 (0.58)	0.73 (0.61)	0.56 (0.56)
College graduate	1.55 (9.61)	0.51 (0.50)	0.69 (0.56)	0.44 (0.45)
No college	31.73 (67.67)	0.72 (0.67)	0.95 (0.64)	0.63 (0.67)
Any college	7.25 (27.37)	0.55 (0.53)	0.71 (0.58)	0.47 (0.49)
Parity				
0	8.12 (29.01)	0.56 (0.58)	0.77 (0.66)	0.51 (0.55)
1	14.86 (45.80)	0.58 (0.54)	0.68 (0.55)	0.53 (0.53)
2	21.66 (49.32	0.62 (0.62)	0.84 (0.62)	0.46 (0.58)
3	34.58 (79.11)	0.71 (0.56)	0.80 (0.52)	0.67 (0.59)
≥4	46.70 (82.56)	0.75 (0.59)	1.01 (0.59)	0.53 (0.50)

Note. Non-smokers identified by cotinine values ≤3 ng/mL. Non-transformed cotinine values are presented. GED = General Equivalency Degree.

**Table 3:** Comparison of self-report passive exposure to cotinine values.

	Self-Reported Passive Exposure		
	No Exposure	Passive Exposure	Total
Cotinine values			
No Exposure (<1.0 ng/mL)	382	50	432
Passive Exposure (1–3 ng/mL)	51	20	71
Total	433	70	503

Note. Sample includes non-smokers identified by cotinine values ≤3 ng/mL who had provided self-report information about passive exposure at home and/or work.
